# 506 Hidradenitis Suppurativa Reddit Support Group: Finding New Meaning in Social Media during the COVID-19 Pandemic

**DOI:** 10.1093/jbcr/irac012.137

**Published:** 2022-03-23

**Authors:** Lekha Yesantharao, Rachana Suresh, Carrie A Cox, Sheera F Lerman, Julie Caffrey

**Affiliations:** Johns Hopkins School of Medicine, Baltimore, Maryland; Johns Hopkins Bloomberg School of Public Health, Baltimore, Maryland; Johns Hopkins Bayview Medical Center, Baltimore, Maryland; Johns Hopkins School of Medicine, Baltimore, Maryland; Johns Hopkins, Baltimore, Maryland

## Abstract

**Introduction:**

Hidradenitis suppurativa (HS) is a chronic inflammation of sweat glands that can result in abscesses and scarring, significantly impacting quality of life. Online support groups provide a platform to connect with other HS patients – increasingly important with pandemic-related social isolation. The popular social media site Reddit allows users with common interests, like HS, to form a community and share information. This study characterizes HS patients’ use of Reddit and social media more broadly before and during the COVID-19 pandemic.

**Methods:**

This study consisted of a cross-sectional survey of HS patients treated at our institution between May 2021 and July 2021, collecting data on patient demographics, HS status, and social media support group usage/interest. A longitudinal analysis of use of a popular HS support page on Reddit from January 2019 to August 2021 was also conducted, analyzing the number of subscribers over time.

**Results:**

The number of subscribers to the subreddit r/Hidradenitis increased exponentially from January 2019 to August 2021 (*R*^*2*^*= 0.9978 *for exponential model fit to data); this suggests that the onset of the COVID-19 pandemic was associated with a greater increase in the number of subreddit subscribers. Further, 20 patients (90% female, mean age of 32.4 years) completed the survey that was administered. Participants were stratified into two groups: online support group users (n=8) and non-users (n=12). There were no significant differences in sex, age, education level, HS activity, antidepressant usage, and overall social media usage between these groups. However, there was a significant difference in Hurley staging between the two groups; 75% (n=6) of online support group users reported a Hurley III staging, while only 16.7% (n=2) of non-users self-reported as Hurley III (*p=0.003*). In terms of features patients desired to see in online support groups, non-users ranked the following categories of advice/information as important more frequently than current users: bandaging/dressing boils, living with HS, medical advice from professionals, causes of HS, and diet (*p=0.047, p=0.043, p=0.043, p=0.047, *and *p=0.013, *respectively).

**Conclusions:**

This study demonstrates that online support group use is associated with patients with HS of higher clinical severity. Since virtual support groups have an unprecedented importance due to increased social isolation and limited access to in-person support groups and health resources, healthcare providers may encourage non-users to partake in these online support communities during these trying times. Based on the needs and expectations of these patients as identified in this study, recommendations can be made to moderators of online communities to help fill any existing lacunae.

